# Fabrication of Diamond Submicron Lenses and Cylinders by ICP Etching Technique with SiO_2_ Balls Mask

**DOI:** 10.3390/ma12101622

**Published:** 2019-05-17

**Authors:** Zongchen Liu, Tian-Fei Zhu, Yan-Feng Wang, Irfan Ahmed, Zhangcheng Liu, Feng Wen, Xiaofan Zhang, Wei Wang, Shuwei Fan, Kaiyue Wang, Hong-Xing Wang

**Affiliations:** 1 Institute of Wide Bandgap Semiconductors, Xi’an Jiaotong University, Xi’an 710049, China; zongchenliu@163.com (Z.L.); zhutianfei2@163.com (T.-F.Z.); yanfengwang@stu.xjtu.edu.cn (Y.-F.W.); liuzhangcheng@stu.xjtu.edu.cn (Z.L.); fengwen@mail.xjtu.edu.cn (F.W.); xiaofan.z@xjtu.edu.cn (X.Z.); wei_wang2014@mail.xjtu.edu.cn (W.W.); shwfan@xjtu.edu.cn (S.F.); wangkaiyue8@163.com (K.W.); 2 Key Lab for Physical Electronics and Devices of the Ministry of Education, School of Electronics and Information Engineering, Xi’an Jiaotong University, Xi’an 710049, China; 3 Department of Electrical Engineering, Sukkur IBA University, Sukkur 65200, Pakistan; ifffy_84@hotmail.com; 4 School of Materials Science and Engineering, Taiyuan University of Science and Technology, Taiyuan 030024, China

**Keywords:** diamond, NV center, microstructure fabrication, micro-optical devices

## Abstract

Submicron lenses and cylinders exhibiting excellent properties in photodetector and quantum applications have been fabricated on a diamond surface by an inductively-coupled plasma (ICP) etching technique. During ICP etching, a layer containing 500 nm diameter balls of SiO_2_ was employed as mask. By changing the mixing ratio of O_2_, Ar and CF_4_ during ICP etching, several submicron structures were fabricated, such as cylinders and lenses. The simulation results demonstrated that such submicron structures on a diamond’s surface can greatly enhance the photon out-coupling efficiency of embedded nitrogen-vacancy center.

## 1. Introduction

The negatively charged nitrogen-vacancy (NV^−^) center in diamond emitting single photon with 637 nm wavelength has appeared as a promising quantum light source [1]. In the meantime, NV^−^ center in diamond has attracted significant interest because of its outstanding properties, such as optical spin-polarization and readout of ground state spin, long coherent time of ground state spin, and flexibility in its device fabrication [1,2,3,4,5]. Since then, NV^−^ center has been employed in a variety of high resolution and highly sensitive detectors used in several application fields, such as biology [6], and for the measurement of physical quantities, such as magnetic field [7], temperature [8] and electrical conductivity [9,10]. On the other hand, diamond has appeared as a promising material for photonic and high-power electronic devices, even in extreme environments due to its excellent properties, such as wide bandgap (5.5 eV), high critical breakdown field (>10 MV·cm^−1^), high thermal conductivity (22 W·cm^−1^·K^−1^), high carrier mobility (~3800 cm^2^·V^−1^·s^−1^ for holes and ~4500 cm^2^·V^−1^·s^−1^ for electrons), and high saturation velocity (10^7^ cm·s^−1^). [11,12] However, the performances of NV^−^ center in applications and diamond photonic devices are limited by low photon in- and out-coupling efficiency [13,14,15].

It is well known that micro-structures can significantly improve the photon in- and out-coupling efficiency, such as micro lenses [16,17] and micro-cylinders [18,19]. Recently, some experiment schemes were performed to fabricate micro lenses on a diamond surface to improve photo response of photodetectors and the efficiency of embedded NV^−^ center [16,17,20]. These works have greatly improved the developments of diamond photodetectors and applications of NV^−^ center. For instance, focus ion beam (FIB) technique has been used to fabricate solid immersion lens (SIL) in several micron sizes [17,21,22]. Photolithography and ICP etching techniques had been used to fabricate micro lenses whose sizes were limited by resolution of photolithography [16,20,23]. Electron beam lithography or dual mask technique and ICP etching techniques were used to fabricate micro cylinders [19,24,25]. However, such micro lenses either have large radius of curvature (ROC) on account of hardness and chemical inertness of diamond or have complex fabrication procedures, for example the FIB technique or dual-mask method was employed. Moreover, it is not easy to fabricate lenses at submicron level using these methods.

Here, a novel and convenient approach is demonstrated to fabricate a large number of diamond submicron lenses and cylinders by employing an inductively-coupled plasma (ICP) etching technique with a resistant mask of economical 500 nm diameter balls of SiO_2_. By changing the reactive gas component and etching time through the ICP etching process, submicron structure with different dimensions were obtained. The finite-difference time-domain (FDTD) method was used to obtain photon out-coupling efficiency of embedded NV^−^ center in submicron structures.

## 2. Methods and Fabrication

**Figure 1:**
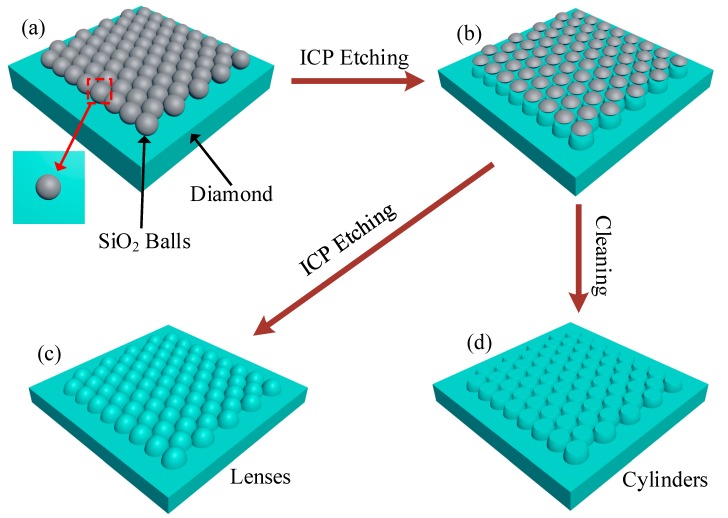
Fabrication process flow of submicron lenses and cylinders. (**a**) Coating a layer of SiO_2_ balls on diamond surface. (**b**) ICP Etching SiO_2_ and diamond simultaneously. (**c**) Lenses were obtained by etched SiO_2_ completely. (**d**) Cylinders were obtained by removing SiO_2_.

**Figure 2:**
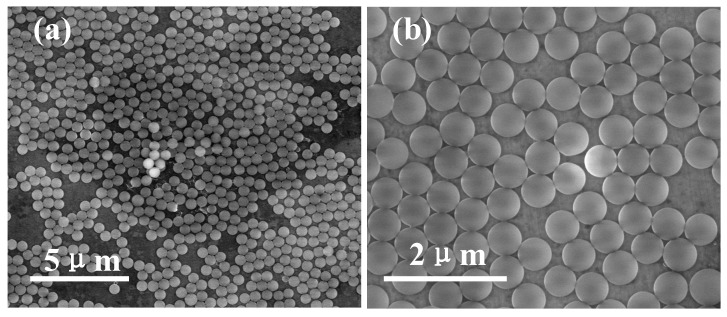
(**a**) SEM of diamond with a layer of 500 nm diameter balls of SiO_2_. (**b**) Magnification of (**a**).

**Figure 3:**
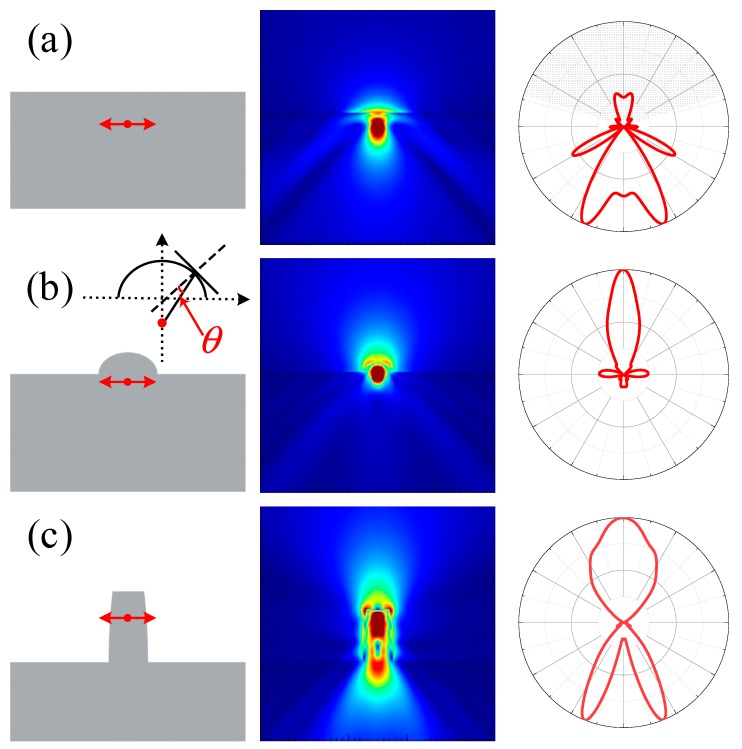
Simulation of electric field and far field of NV^−^ center embedded in submicron structures. (**a**) NV^−^ center in bulk diamond; (**b**) NV^−^ center in micron lens with 500 nm diameter; inset, diagram of light propagation in lens. (**c**) NV^−^ center in submicron cylinder. The first column demonstrates the positions of NV^−^ center; the second column presents the corresponding electric field simulations; the third column presents the corresponding far-fields of NV^−^ center in which the light grey shadows are the regions that can be detected by microscopy with NA of 0.95.

**Figure 4:**
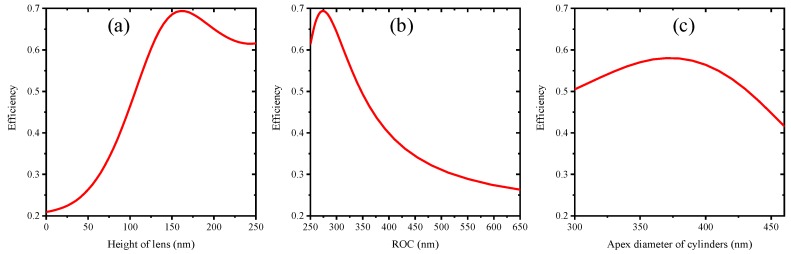
Photon collection efficiency of NV^−^ center versus submicron structure dimensions. (**a**) efficiency versus lenses heights when diameters of lenses are 500 nm; (**b**) efficiency versus ROC of submicron lenses; (**c**) efficiency versus apex diameter of submicron cylinders.

Figure 1 shows the fabrication process of submicron structures. Type Ib (100) diamond substrates were cleaned in acid solution (HCl:HNO_3_ = 3:1 by volume) at temperature of 80 °C for 30 min prior to experiment. SiO_2_ balls (S2-00500, CV% = 3–5%, Tianjin DAE Scientific Co. Ltd, Tianjin, China) were coated on diamond surface by using dip-coating method and employed as ICP etching mask. At first, SiO_2_ suspension (2.5 wt.% in water) was ultrasonicated for 5 min. Following this treatment, diamond substrate was immersed in suspensions for ten seconds and was pulled up followed by drying at 100 °C. As a result, a layer of SiO_2_ balls with 500 nm diameter was coated on diamond substrate as shown in Figure 2. Then, diamond with coated SiO_2_ balls were etched simultaneously by ICP etching technique as shown in Figure 1b. During etching, the ICP power and RF power were fixed at 450 W and 50 W, respectively. By controlling ICP reactive gas component and etching time, several submicron structures were obtained on the diamond surface. By using Lumerical FDTD Solutions, simulations were performed to obtain and optimize photon collection efficiency of NV^−^ centers embedded in bulk diamond, submicron lenses and submicron cylinders. The simulation results were shown in Figure 3 and Figure 4.

In order to transfer the curvature of the balls to the diamond surface as shown in Figure 1c, SiO_2_ balls and diamond should be etched simultaneously. Therefore, a mixture gas of CF_4_ and O_2_ was used during ICP etching, which reacts with both SiO_2_ and diamond. During this process of etching, etching time, ICP power, RF power and O_2_ flow rate were fixed at 5 min, 450 W, 50 W and 50 sccm, respectively. Submicron lenses with several heights were obtained by changing ratio of CF_4_ while fixing other ICP etching parameters, which resulted in changing of etching ratio *k* of SiO_2_ to diamond. Relationship between submicron lens height *h* and *k* can be expressed as,

(1) $$h=\frac{d}{k}$$

where *d* = 500 nm is diameter of SiO_2_ balls. *k* can be measured by calculating ratio of mask thickness to structure height. Here, *k* is ratio of SiO_2_ diameter to submicron lens height. The relationship between ROC and lens height can be described as,

(2) $$\mathrm{ROC}=\frac{h^{2}+{\left(d/2\right)}^{2}}{2h}$$

**Figure 5:**
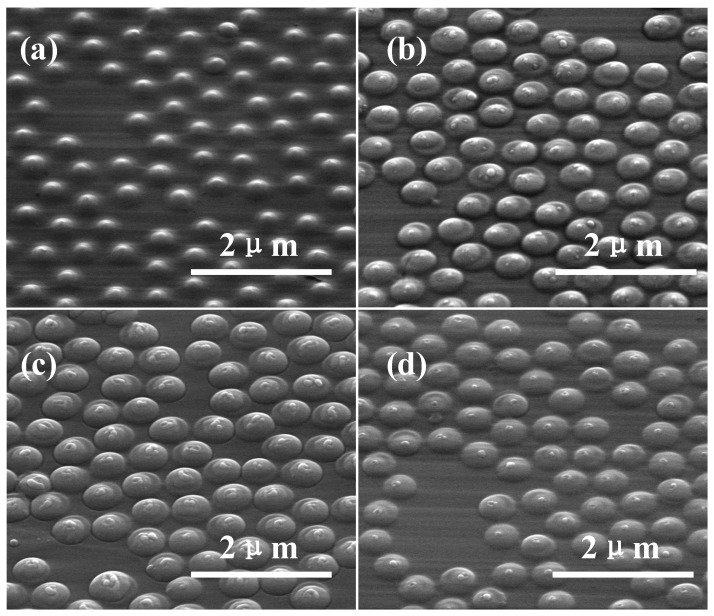
SEM images of submicron lenses on diamond surface acquired with 45° tilt angle of samples. (**a**–**d**) Submicron lenses fabricated by using CF_4_ flow rate of 10, 15, 20 and 25 sccm, respectively.

**Figure 6:**
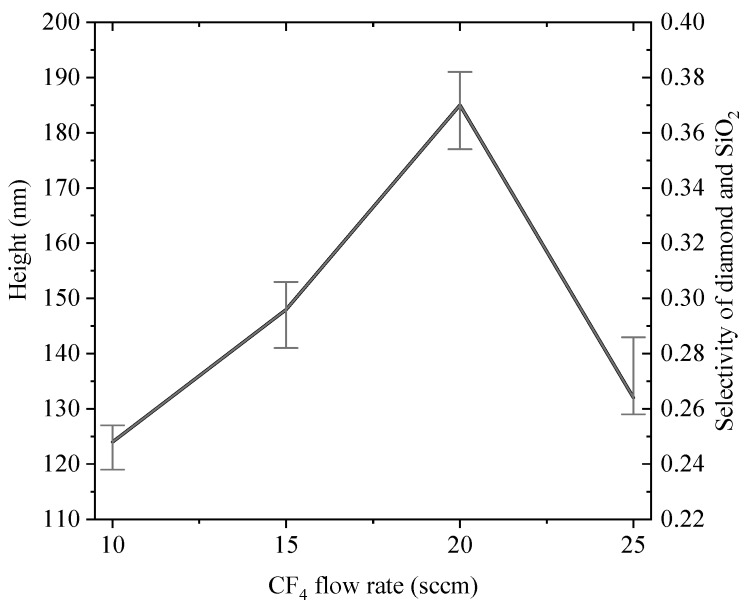
Relationship between submicron lens height and CF_4_ flow rate (left axes), and selectivity of diamond and SiO_2_ as a function of CF_4_ flow rate (right axes).

Figure 5a–d show SEM images of submicron lenses which were fabricated by setting CF_4_ flow rates at 10, 15, 20 and 25 sccm, respectively. Submicron lens heights were obtained by averaging randomly acquired 10 Atomic Force Microscope (AFM) measurements and were presented in Figure 6 (left axes). After ICP etching, the SiO_2_ balls were completely etched by mixture gas of CF_4_ and O_2_, therefore, the selectivity of diamond and SiO_2_ is defined as ratio of lens height and balls diameter of SiO_2_, which were also presented in Figure 6 (right axes). When diameters of lenses are uniform, ROC of lenses is a function of lenses height, higher lens height corresponds to smaller ROC.

**Figure 7:**
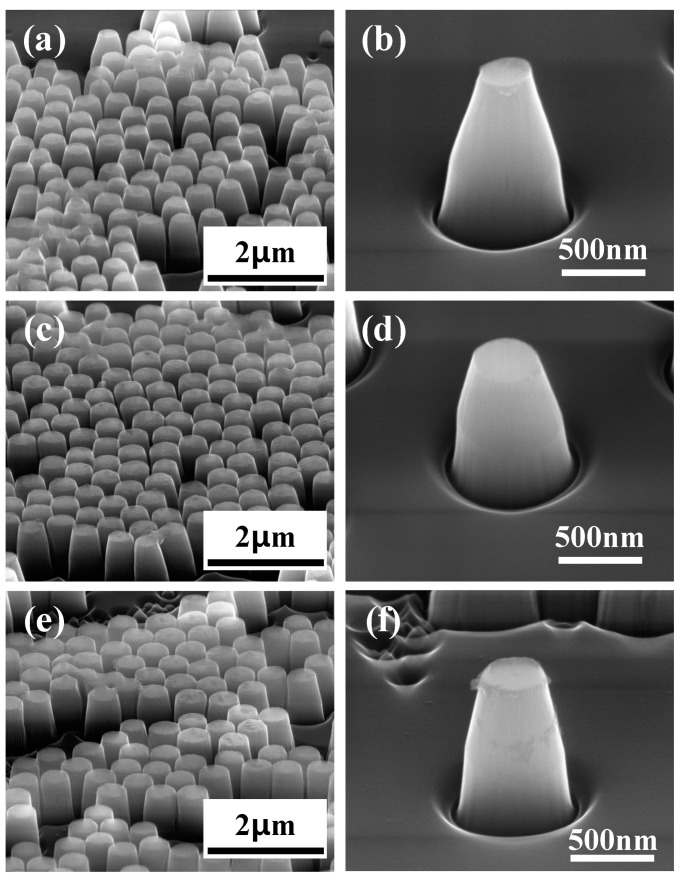
SEM images of cylinders on diamond surface acquired with 45° tilt angle of samples. (**b**,**d**,**f**) are magnified single structure of (**a**,**c**,**e**), respectively. (**a**,**c**,**e**) were fabricated when Ar flow rate was 5, 10 and 15 sccm, respectively.

**Figure 8:**
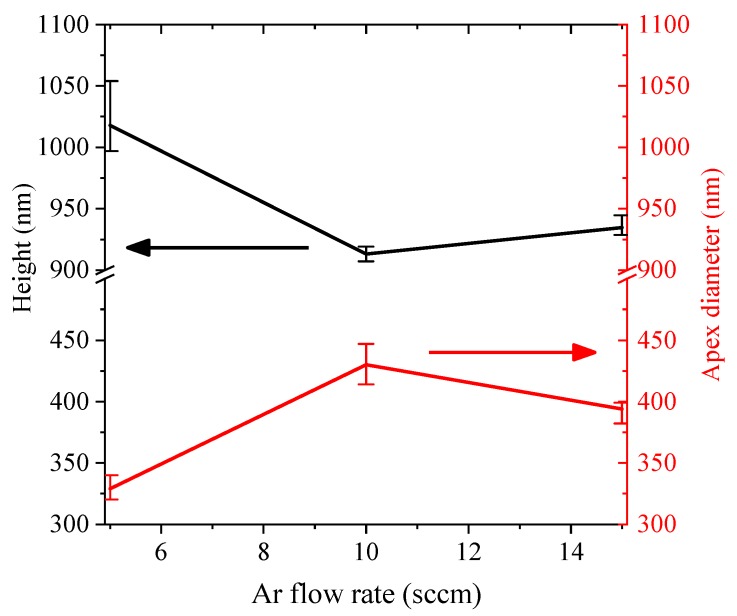
Submicron lens height (black line, left axes) and apex diameter (red line, right axes) versus Ar gas flow rate.

Different to submicron lenses fabrication, the etching speed of SiO_2_ should be slower than diamond when fabricating submicron cylinders. Therefore, CF_4_ was replaced by Ar throughout ICP etching process. Figure 1d shows the cylinder’s case, of which the ICP gas was mixture of O_2_ and Ar. During ICP etching, etching time, ICP power, RF power and O_2_ flow rate were fixed at 10 min, 450 W, 50 W and 50 sccm, respectively. Several cylinders were obtained by changing the ratio of Ar. Finally, the samples were cleaned ultrasonically in acetone and methanol baths to remove residuals of SiO_2_. Figure 7 shows cylinders with different heights and apex diameters (top face diameter of cylinder), whose values were obtained by averaging randomly acquired 10 AFM measurements and were presented in Figure 8.

## 3. Results and Discussion

### 3.1. Simulation and Optimization

NV^−^ center can emit photons of 637 nm wavelength by laser excitation. Figure 3 shows electric field (second column) and far-field (third column) of NV^−^ centers, while the light gray shadow in far-field is the region that can be collected by microscopy with numerical aperture (NA) of 0.95. Figure 3a shows that most of photons emitted from NV^−^ center cannot escape from bulk diamond because of total internal reflection, indicating limitation of photon collection efficiency up to 20.9% only. To improve this, submicron lenses and submicron cylinders have been added above NV^−^ center on diamond surface. Figure 3b shows a submicron lens with diameter of 500 nm above NV^−^ centers on diamond surface, this suggests most photons emitted from NV^−^ center can escape from diamond and can be detected by optical detection system.

Figure 4a,b shows NV^−^ center photon collection efficiency as a function of 500 nm diameter lens height and lens ROC, respectively. This demonstration suggests that the efficiency was at maximum when the height (ROC) is 160 nm (275 nm). The efficiency became lower if we continued increasing the height of submicron lens after 160 nm, however, the efficiency was still more than 60%. On the other hand, submicron cylinders with a curving side wall can also improve photon collection efficiency effectively as suggested and depicted in Figure 3c. The photon collection efficiency of submicron cylinders with 500 nm diameter and 950 nm height as a function of apex diameter has been shown in Figure 4b. The reflective index of diamond is about 2.42, which corresponds to 24.4° of critical angle of total reflection. Increasing lens height will increase *θ* as shown in the inset of Figure 3b, when *θ* is larger than 24.4°, the emission of NV^−^ center cannot escape from the diamond in this direction. Therefore, the photon collection efficiency attains maximum value when lens height was increased. Similarly, efficiency of cylinders also attains maximum value when apex diameter was changed.

### 3.2. Submicron Lenses

In this section, submicron lenses with several heights were obtained by controlling etching ratio of SiO_2_ and diamond as shown in Figure 5. As indicated in Figure 6, lenses’ height increased by improving CF_4_ flow rate up to 20 sccm. According to Equation (1), we can infer that etching ratio *k* gradually decreases with CF_4_ flow rate goes up to 20 sccm. When CF_4_ flow rate reaches 25 sccm, etching ratio *k* suddenly appears to be improved, which leads to a decrease of lens height. According to Figure 4a, more than 60% photon can be collected by the optical system when lens height is above 120 nm. Therefore, such fabricated lenses can improve collection efficiency by at least 3 times.

### 3.3. Submicron Cylinders

The submicron cylinders also can greatly improve photon collection efficiency of embedded NV^−^ center. Figure 7a,c,e correspond to 5, 10 and 15 sccm Ar gas flow rates, respectively. Trenches appeared around the bottom of cylinder; this is because of the deflection of accelerated ions along the already etched side walls of the cylinders [16]. A SiO_2_ ball in the top view of sample is the circle which is displayed in the inset of Figure 1a. The circle’s edge thickness is thinner than the middle position. Therefore, one can predict that the edge of this circle will be etched faster than middle position, while the diameter of this circle decreases as etching time goes on. Meanwhile, the diameter of cylinder apex also decreases as etching time goes on because it is same as the diameter of circle. Therefore, the diameter of bottom part is larger than that of apex. By using different etching parameters, the final diameters of these circles were different, which induced different apex diameters of cylinders.

The height of cylinder reflects the etching speed of diamond because the top faces of cylinders have not been etched. Meanwhile, apex diameter of cylinder reflects etching speed of SiO_2_. Large height and small apex diameter suggest high etching speed of diamond and SiO_2_. It can be predicted from Figure 8 that etching speed of diamond and SiO_2_ are hugely decreased when Ar flow rate increased from 5 sccm to 10 sccm. When Ar flow rate increases from 10 sccm to 15 sccm, the etching speed of diamond and SiO_2_ were observed with slight increment. Therefore, by changing Ar flow rate during ICP etching, submicron cylinders with several apex diameters and heights were obtained, which can greatly improve photon collection of embedded NV^−^ centers.

## 4. Conclusions

Submicron lenses and cylinders were fabricated on a diamond surface by using the ICP etching technique with a resistant mask of a layer of 500 nm diameter SiO_2_ balls. By changing the mixing ratio of CF_4_ and O_2_ gases during the ICP etching process, several heights of submicron lenses with diameters of 500 nm were obtained. Be employing a mixture gas of Ar and O_2_, submicron cylinders with several apex diameters were obtained with height of about 1 μm. The simulation results demonstrated that fabricated submicron structures can significantly improve photon collection efficiency of embedded NV^−^ center at least by threefold when compared with that of NV^−^ center in bulk diamond. These submicron structures also have potential to be used in quantum optic and photoelectric detection applications.
